# Assessing Racial Disparities in Guideline-Concordant Care and Clinical Outcomes after Surgical Resection of Nonmetastatic Colon Cancer at a Comprehensive Cancer Center

**DOI:** 10.1158/2767-9764.CRC-24-0633

**Published:** 2025-07-18

**Authors:** Christina I. Lee, Sharafudeen D. Abubakar, Fan Wu, Hannah M. Thompson, Farheen Shah, Michele Waters, Jonathan B. Yuval, Hannah Williams, Anisha Luthra, Dana M. Omer, Chin-Tung Chen, Julio Garcia-Aguilar, Francisco Sanchez-Vega

**Affiliations:** 1 Department of Surgery, Colorectal Service, Memorial Sloan Kettering Cancer Center, New York, New York.; 2 Department of Epidemiology and Biostatistics, Memorial Sloan Kettering Cancer Center, New York, New York.; 3 Tel Aviv Sourasky Medical Center and Tel Aviv University, Tel Aviv, Israel.

## Abstract

In this study, we examined racial disparities in guideline-concordant care (GCC) and clinical outcomes of patients with colon cancer treated at a single comprehensive cancer center. We analyzed data from self-reported Hispanic, non-Hispanic Black (NHB), and non-Hispanic White (NHW) patients who underwent curative colectomy for stage I to III colon cancer between 2006 and 2021 at Memorial Sloan Kettering Cancer Center. GCC was defined as retrieval of ≥12 lymph nodes and appropriate receipt of adjuvant chemotherapy. Recurrence and overall survival from the time of surgery were compared using the Kaplan–Meier method and the log-rank test. Multivariable analyses were performed using Cox regression. The study included 2,209 patients, with 1,911 NHW, 153 NHB, and 145 Hispanic patients. NHW patients were older, whereas NHB patients had higher percentages of Medicaid coverage, obesity, and lower socioeconomic status. NHB patients more often presented with stage III disease and underwent open surgery. Receipt of GCC was not different by race. NHB patients had the highest 5-year recurrence rate compared with NHW and Hispanic patients (27% vs. 15.7% vs. 15.1%; *P* = 0.03). NHB race (HR = 1.43; *P* = 0.07) and low body mass index (HR = 1.98; *P* = 0.05) were associated with an increased risk of recurrence with marginal significance. NHB race was associated with an increased risk of recurrence in stage I disease (HR = 3.52; *P* = 0.03). NHB patients had shorter recurrence-free survival, despite standardized quality of care. NHB race was independently associated with an increased risk of recurrence in stage I disease.

**Significance:**

This study compares receipt of GCC, disease recurrence, and survival among White, Black, and Hispanic patients with nonmetastatic colon cancer treated at a single comprehensive cancer center with standardized quality of care and comparable access to health care. Black patients had higher rates of recurrence in this study.

## Introduction

Colorectal cancer is the third most common cancer and the second most common cause of cancer death in the United States (1). Advances in colorectal cancer surveillance and management have led to a significant overall improvement in recurrence and survival rates. However, racial disparities in care and outcomes for patients with colorectal cancer have been widely described in all steps of care, including screening, diagnosis, surveillance, access to health care, and outcomes (1–3).

Screening colonoscopy resources remain unequally distributed across races, with a lower screening prevalence noted in Hispanic and non-Hispanic Black (NHB) patients (1, 4–6). A study comparing the quality of colonoscopies showed that NHB patients received colonoscopy from physicians with lower polyp-detection rates compared with non-Hispanic White (NHW) patients, which was associated with increased colorectal cancer risk (7). NHB patients were also noted to have higher incidence of colorectal cancer and higher percentage of younger patients presenting with more advanced disease at diagnosis (5, 8). NHB patients have been shown to undergo less treatment, including surgery and chemotherapy, than their NHW counterparts (9–12). Moreover, differential access to health care in the United States, with NHB patients being less often insured than NHW patients, further amplifies the complexity of disparities in colorectal cancer care (13–15). Ultimately, NHB patients have been shown to have significantly worse survival from colorectal cancer compared with NHW patients (16–20).

Racial and ethnic disparities in health care are a result of a complex interplay of a multitude of variables, including socioeconomic and historical factors. Most previous studies investigating these disparities have utilized large nationwide databases or regional registries, in which access to care and quality of treatment are largely variable (4, 16, 18–22). Using such heterogeneous study cohorts may not successfully delineate the intrinsic racial factors driving disparities, given a myriad of confounders (23). We sought to attenuate the potential confounding effect of variability in care across hospitals by examining patients who were treated under uniform guidelines at a high-volume comprehensive cancer center by a set group of colorectal surgeons, oncologists, and radiation oncologists. In addition, all patients in this cohort had health coverage or financial means to afford health care. This eliminates the mediating effect of uninsurance, which is a major barrier to accessing health care and therefore associated with worse outcomes, while still allowing for investigation of the effect of different types of insurance. As such, this cohort allows for the evaluation of disparities in outcomes that persist when quality of treatment is consistent within a single healthcare system.

We analyzed real-world data collected from NHW, NHB, and Hispanic patients who underwent surgical resection for nonmetastatic colon cancer and examined racial and ethnic differences in guideline-concordant care (GCC), as well as oncologic outcomes. Subsequently, we explored potential factors independently driving such disparities while controlling for relevant clinicopathologic and socioeconomic variables.

## Materials and Methods

### Patient selection

**Figure 1:**
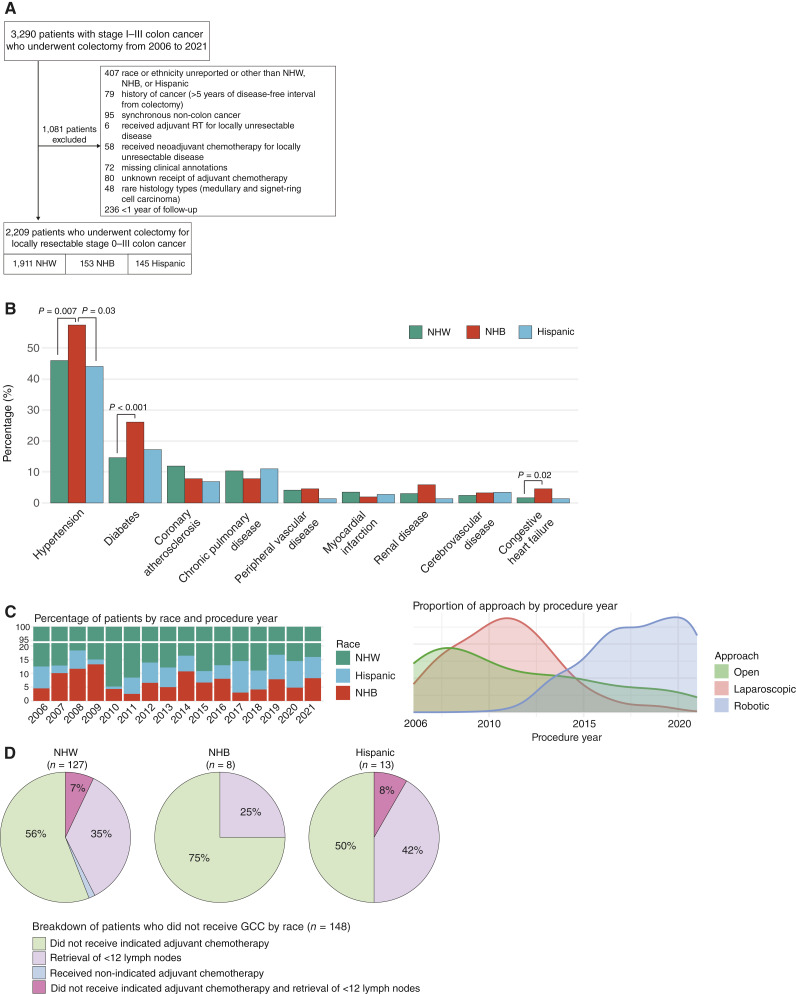
Cohort overview and description of clinical features. **A,** CONSORT diagram of patients included in the study. RT, radiotherapy. **B,** Baseline comorbidities by racial and ethnic groups. **C,** Description of the type of operative approach by racial group and procedure year. Left top plot, percentage of racial groups included in the study by procedure year; left bottom plot, ratio of the approach by procedure year. **D,** Differences in receipt of GCC by racial groups. Results are shown for NHW (left), NHB (middle), and Hispanic (right) patients.

This study was approved by the Institutional Review Board at Memorial Sloan Kettering Cancer Center (MSKCC). Prospectively maintained institutional databases were queried to identify patients with treatment-naïve locally resectable stage I to III colon adenocarcinoma who underwent curative resection at MSKCC between 2006 and 2021. Patients who self-identified as NHW, NHB, or Hispanic were included in this study, and those who self-identified as other combinations of race and ethnicity, or lacked this information, were excluded. Those with a diagnosis of rectal cancer (≤12 cm from the anal verge) or metachronous colon cancer within 5 years prior to their colectomy and those with a history of non-colon cancer (not including nonmelanotic skin cancers) within the 5 years prior to their colon cancer diagnosis were excluded. Those with synchronous rectal or non-colon cancer were also excluded. Patients who did not have locally resectable disease were excluded. Lastly, patients with missing demographic, clinical, and treatment characteristics, as specified below, were excluded (Fig. 1A). Only patients with ≥1 year of follow-up after surgical resection were included in the study.

All patients underwent curative resection. Operative approach (open, laparoscopic, or robotic surgery), extent of resection, method of anastomosis, and extraction site depended on the surgeon’s preference. In order to evaluate for resection quality, total lymph node yield and retrieval of ≥12 lymph nodes were obtained from the pathology reports. Staging was determined based on the eighth edition of the American Joint Committee on Cancer (AJCC) guidelines (24). Patients underwent adjuvant chemotherapy and further surveillance according to the recommendations of their treating medical oncologist and surgeon.

### Collection and curation of demographic and clinical information

Data for clinically relevant variables were manually extracted from the electronic medical record. Baseline demographic variables including sex, age at diagnosis, history of cancer (>5 years of disease-free interval from colectomy), body mass index (BMI), and American Society of Anesthesiologists (ASA) scores were collected. Treatment and tumor characteristics including surgical approach, readmission within 90 days after surgery, tumor location, tumor histology, tumor grade, mismatch repair (MMR) status, and pathologic AJCC overall stage were collected. For tumor location, tumors involving the cecum, ascending colon, hepatic flexure, and transverse colon were categorized as right-sided, whereas tumors involving the splenic flexure, descending colon, and sigmoid colon were categorized as left-sided. Criteria used to define GCC for nonmetastatic colon cancer included retrieval and examination of ≥12 regional lymph nodes and initiation of adjuvant chemotherapy for patients with pathologic high-risk T3–4Nx or any TxN1–2 disease, as specified in the National Comprehensive Cancer Network treatment guidelines (25).

### Collection and curation of socioeconomic information

Social factors including insurance type and Yost index were obtained. Insurance type was identified and dichotomized into Medicaid and non-Medicaid (which included commercial, Medicare, and self-pay), based on socioeconomic considerations. Neighborhood socioeconomic status (SES) was quantified using the Yost index, which is a composite measure computed across geographic areas and integrates information about household income, poverty, rent, house value, education, employment type, and employment status, from the American Community Survey of the United States census (26, 27). Yost index values range between 1 and 100, with higher scores representing lower SES. Specifically, the Yost index was computed for each patient in the study at the census-tract level based on their most recent billing address on file. Approximately 26% of values were imputed by taking the median of the Yost indexes of the corresponding patient zip code. Furthermore, patients were categorized into high, medium, and low SES based on their Yost indexes with the Jenks natural breaks classification method using the classlnt package in R, which divides a linear dataset into groups or classes to minimize variance within each group while maximizing variance between groups (28). Yost index values of 0 to 22.3 were categorized as high, 22.3 to 52 as medium, and >52 as low SES in this cohort.

### Clinical outcomes

Recurrence of a patient’s primary colon cancer was defined as the presence of radiological and/or pathologic evidence of either local and/or distant recurrence. Overall survival (OS) accounted for deaths from any cause and was calculated from the date of surgery. The date of last follow-up was used to censor patients.

### Statistical analyses

Categorical variables are presented as frequencies and percentages and compared using the Fisher exact test. The Kaplan–Meier or product-limit method was used to visualize time to recurrence and OS, and groups were compared using the log-rank test. Multivariable analyses using Cox proportional hazards models were performed to identify predictors of recurrence and survival. Statistical significance was defined as a *P* value of < 0.05. All analyses were performed using R software version 4.1.1 (R Core Team, RRID: SCR_001905).

### Data availability

The data generated in this study are available within the article and its supplementary data files.

## Results

Between January 2006 and December 2021, 3,290 patients with stage I to III colon cancer underwent colectomy at MSKCC. After exclusion of 1,081 patients as outlined in Fig. 1A, 2,209 patients were included for analysis, consisting of 1,911 (86.5%) NHW, 153 (6.9%) NHB, and 145 (6.6%) Hispanic patients. Outcome data were last updated on February 1, 2025. The median follow-up for the entire cohort was 5.12 years, and the specific median follow-ups for each group were 5.14, 5.11, and 4.82 years for NHW, NHB, and Hispanic patients, respectively (*P* = 0.15). Throughout the entire follow-up period, there were a total of 330 recurrence events and 480 deaths. All the clinical information used in our analyses is provided in Supplementary Table S1.

A summary of baseline patient features stratified by race is provided in Table 1. The median age of the cohort at diagnosis was 64 years (IQR, 53–74), and 49% (1,081/2,209) were women. The median age at diagnosis was lower in NHB (61 years; IQR, 53–67) and Hispanic patients (61 years; IQR, 52–73) compared with NHW patients (64 years; IQR, 53–74; *P* = 0.02). NHB patients also included a significantly larger proportion of middle-aged patients compared with NHW and Hispanic patients (52% vs. 33% and 40%; *P* < 0.001). NHB patients also had a higher proportion of obese patients (51% vs. 38% and 36% in NHW and Hispanic patients, respectively; *P* = 0.033). No significant differences were observed by race in terms of sex, history of cancer >5 years in remission, ASA physical status score, and general presence or absence of comorbidities within this cohort. When specific comorbidities were examined, however, NHB patients had significantly higher percentages of hypertension compared with NHW (58% vs. 46%; *P* = 0.007) and Hispanic patients (58% vs. 44%; *P* = 0.03). NHB patients also had significantly higher percentages of diabetes (26% vs. 15%; *P* < 0.001) and congestive heart failure (4.6% vs. 1.7%; *P* = 0.02) compared with NHW patients (Fig. 1B).

**Table 1:** Summary of demographics

Characteristic	Hispanic (*N* = 145)	NHB (*N* = 153)	NHW (*N* = 1,911)	*P* value
Age, years, *n* (%)	​	​	​	<0.001
<50	26 (18%)	20 (13%)	332 (17%)	​
50–64	58 (40%)	80 (52%)	627 (33%)	​
>65	61 (42%)	53 (35%)	952 (50%)	​
Sex, *n* (%)	​	​	​	0.10
Female	79 (54%)	84 (55%)	918 (48%)	​
Male	66 (46%)	69 (45%)	993 (52%)	​
History of cancer, *n* (%)	9 (6.2%)	9 (5.9%)	197 (10%)	0.074
ASA score, *n* (%)	​	​	​	0.7
1	1 (0.7%)	3 (2.0%)	15 (0.8%)	​
2	58 (40%)	55 (36%)	710 (37%)	​
3	81 (56%)	89 (58%)	1,126 (59%)	​
4	5 (3.4%)	6 (3.9%)	60 (3.1%)	​
Comorbidities, *n* (%)	​	​	​	0.10
Absent	67 (46%)	54 (35%)	836 (44%)	​
Present	78 (54%)	99 (65%)	1,075 (56%)	​
BMI, *n* (%)	​	​	​	0.033
Underweight	4 (2.8%)	0 (0%)	26 (1.4%)	​
Normal	37 (26%)	31 (20%)	510 (27%)	​
Overweight	52 (36%)	44 (29%)	642 (34%)	​
Obese	52 (36%)	78 (51%)	733 (38%)	​
^a^nSES category, *n* (%)	​	​	​	<0.001
Low SES	38 (26%)	55 (36%)	241 (13%)	​
Middle SES	53 (37%)	58 (38%)	525 (27%)	​
High SES	54 (37%)	40 (26%)	1,145 (60%)	​
Insurance, *n* (%)	​	​	​	<0.001
Non-Medicaid	141 (97.2%)	138 (90%)	1,859 (93.3%)	​
Medicaid	4 (2.8%)	15 (9.8%)	52 (2.7%)	​

Abbreviations: ASA, American Society of Anesthesiologists; nSES, neighborhood socioeconomic status.

a Low, middle, and high categories were defined as described in the Methods section.

Socioeconomic features were compared across racial and ethnic groups (Table 1). NHB patients had the highest median Yost index (41; IQR, 22–62), followed by Hispanic (30; IQR, 16–56) and NHW patients (18; IQR, 7–34; *P* < 0.001). Patients were then classified into low-, medium-, and high-SES groups based on their Yost index using the Jenks natural breaks classification method as described above. NHB patients had the largest proportion of low SES and lowest proportion of high SES compared with the other two groups (*P* < 0.001). NHB patients were found to have a higher percentage of Medicaid coverage compared with NHW and Hispanic patients (9.8% vs. 2.8% and 2.7%; *P* < 0.001).

A summary of tumor and treatment characteristics is provided in Table 2. NHB patients had a higher percentage of open colectomy compared with NHW and Hispanic patients (31% vs. 23% and 23%; *P* = 0.015). This can partially be explained by a higher proportion of NHB patients being accrued in the early study period (2006–2009), when more open surgeries were done compared with minimally invasive approaches (Fig. 1C). No difference in the frequency of readmission within 90 days of surgery was observed among the three racial and ethnic groups. Although NHB patients had a higher percentage of right-sided tumors compared with Hispanic and NHW patients, this difference was not statistically significant (63% vs. 54% vs. 55%; *P* = 0.14). In terms of grade, well-differentiated and moderately differentiated tumors were grouped together for analysis because of the small number of well-differentiated cases (*n* = 39). Although the majority of the cases were well-differentiated or moderately differentiated adenocarcinomas, NHW patients had a significantly higher proportion of poor differentiation (18% vs. 9.7% vs. 11%; *P* = 0.003) and mucinous adenocarcinoma (5.4% vs. 2.6% vs. 1.4%; *P* = 0.032) compared with Hispanic and NHB patients. A total of 1,624 (73.5%) patients had documented MMR status based on IHC staining. No significant differences in the proportion of MMR deficiency were observed across NHW (331/1,400; 24.6%), NHB (24/107; 22.4%), and Hispanic patients (23/117; 19.7%; *P* = 0.6). Despite higher percentages of aggressive histology and higher grade in NHW patients, NHB patients had a higher percentage of stage III disease compared with NHW and Hispanic patients (44% vs. 34% and 40%; *P* = 0.02).

**Table 2:** Treatment and tumor-related features

Characteristic	Hispanic (*N* = 145)	NHB (*N* = 153)	NHW (*N* = 1,911)	*P* value
Approach, *n* (%)	​	​	​	0.015
Open	34 (23)	47 (31)	437 (23)	​
Laparoscopic	26 (18)	36 (24)	533 (28)	​
Robotic	85 (59)	70 (46)	941 (49)	​
90-day readmission, *n* (%)	9 (6.2)	9 (5.9)	141 (7.4)	0.8
Tumor site, *n* (%)	​	​	​	0.14
Left-sided	66 (46)	56 (37)	855 (45)	​
Right-sided	79 (54)	97 (63)	1,056 (55)	​
Histology, *n* (%)	​	​	​	0.032
Adenocarcinoma	143 (99)	149 (97)	1,807 (95)	​
Mucinous adenocarcinoma	2 (1.4)	4 (2.6)	104 (5.4)	​
Grade, *n* (%)	​	​	​	0.003
Well/moderately differentiated	131 (90)	136 (89)	1,562 (82)	​
Poorly differentiated	14 (9.7)	17 (11)	349 (18)	​
MMR status, *n* (%)	​	​	​	0.2
Deficient	23 (16)	24 (16)	331 (17)	​
Proficient	94 (65)	83 (54)	1,069 (56)	​
Unknown	28 (19)	46 (30)	511 (27)	​
Pathologic stage, *n* (%)	​	​	​	0.020
I	29 (20)	41 (27)	533 (28)	​
II	58 (40)	44 (29)	722 (38)	​
III	58 (40)	68 (44)	656 (34)	​

A summary of GCC receipt percentages, broken down by nodal retrieval and adjuvant chemotherapy, is described in Table 3. Overall, 97.2% (2,147/2,209) patients had ≥12 lymph nodes removed during colectomy and 95.7% (2,114/2,209) received guideline-concordant adjuvant chemotherapy. No differences in nodal retrieval, guideline-concordant adjuvant chemotherapy, and overall GCC were observed among the racial and ethnic groups. The breakdown of the specific criteria for not meeting guideline recommendations is described in Fig. 1D.

**Table 3:** Summary of receipt of GCC by racial groups

Criteria	Hispanic, *N* = 145 (%)	NHB, *N* = 153 (%)	NHW, *N* = 1,911 (%)	*P* value
Nodal retrieval		​	​	0.3
GCC (≥12 lymph nodes)	139 (95.9)	151 (98.7)	1,857 (97.2)	​
Non-GCC (<12 lymph nodes)	6 (4.1)	2 (1.3)	54 (2.8)	​
Adjuvant chemotherapy^a^		​	​	>0.9
GCC	138 (95.2)	147 (96.1)	1,829 (95.7)	​
Non-GCC	7 (4.8)	6 (3.9)	82 (4.3)	​
Overall		​	​	0.4
GCC	132 (91)	145 (94.8)	1,784 (93.4)	​
Non-GCC	13 (9)	8 (5.2)	127 (6.6)	​

a GCC for adjuvant chemotherapy was defined as (i) adjuvant chemotherapy for stage III, (ii) adjuvant chemotherapy for stage II with high-risk features (<12 lymph nodes retrieved and evidence of lymphovascular invasion), and (iii) no adjuvant chemotherapy for stage I and low-risk stage II. Non-GCC was defined as (i) no adjuvant chemotherapy for stage III and stage II with high-risk features or (ii) adjuvant chemotherapy for stage I and low-risk stage II disease.

**Figure 2:**
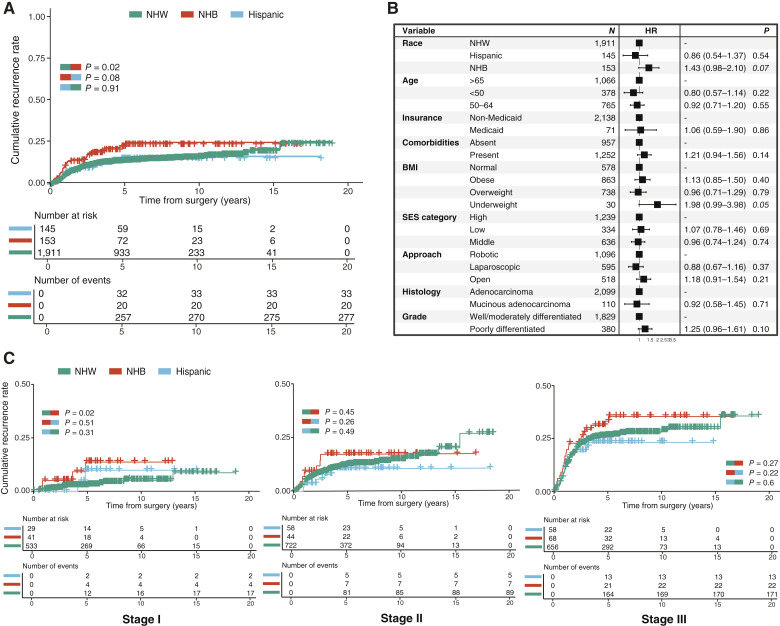
Comparison of clinical outcomes after surgical resection for NHW, NHB, and Hispanic patients. **A,** Kaplan–Meier (product-limit) estimator shows cumulative recurrence rates. The log-rank test was used to compare recurrence rates of NHW, NHB, and Hispanic patients. **B,** Forest plot shows results from multivariate Cox regression analysis for recurrence. **C,** Kaplan–Meier (product-limit) estimators show stage-specific cumulative recurrence rates.

For the overall cohort, 5- and 10-year recurrence rates were 15.8% and 17.3%, whereas 5- and 10-year OS rates were 87.5% and 66.9%. NHB patients had earlier recurrence compared with NHW (*P* = 0.02) and Hispanic (*P* = 0.08) patients (Fig. 2A). NHB patients had significantly higher 5-year recurrence rates (23.1% vs. 15.6% vs. 15.8%; *P* = 0.03) and 10-year recurrence rates (24.2% vs. 16.9% vs. 15.8%; *P* = 0.03) than NHW and Hospanic patients.

Multivariable analyses were performed to identify predictors of recurrence. Variables significantly associated with increased risks of recurrence in univariable analyses (Supplementary Fig. S1) or different across the three racial and ethnic groups (Table 1) were included in the multivariable models. Pathologic AJCC stage and GCC were stratified within the Cox regression model in order to meet the proportional hazards assumption (Fig. 2B). NHB race [HR = 1.43; 95% confidence interval (CI), 0.98–2.10; *P* = 0.07] and BMI <18.5 (HR = 1.98; 95% CI, 0.99–3.98; *P* = 0.05) were associated with an increased risk of recurrence with marginal significance. No significant differences in OS were observed across the three racial and ethnic groups (*P* = 0.25, Supplementary Fig. S2A), and NHB race was not an independent factor associated with an increased risk of death (Supplementary Fig. S2B). The presence of comorbidities was the only independent factor associated with an increased risk of death (HR = 1.91; 95% CI, 1.59–2.43; *P* < 0.001), whereas younger age was associated with a decreased risk of death (HR = 0.23; 95% CI, 0.15–0.35; *P* < 0.001).

Analyses of stage-specific survival by racial and ethnic groups were performed (Fig. 2C). NHB patients had earlier and higher rates of recurrence in general, but significance was only observed in stage I disease when compared with NHW patients (*P* = 0.02). No statistically significant differences in recurrence across the racial and ethnic groups were noted in stage II or III cases. In stage-specific multivariable analyses, NHB race was independently associated with an increased risk of recurrence only in stage I disease (HR = 3.52; 95% CI, 1.12–11.0; *P* = 0.03), and poor differentiation was associated with an increased risk of recurrence in stage III disease (HR = 1.47; 95 CI, 1.08–2.00; *P* = 0.01; Supplementary Fig. S2C). No independent factor was associated with a risk of recurrence in stage II disease.

## Discussion

We present one of the largest single-institution studies to date comparing racial and ethnic disparities in oncologic outcomes among NHW, NHB, and Hispanic patients after surgical resection of nonmetastatic colon cancer. As the mortality rate associated with colon cancer has been consistently decreasing over the years and the number of early-onset colon cancer cases is on the rise, accurate collection of recurrence data is quickly becoming an important measure for assessing the disease burden within the colon cancer population.

Our data demonstrate that NHB patients experienced earlier recurrence of disease and had higher recurrence rates compared with NHW and Hispanic patients. After controlling for clinicopathologic and socioeconomic differences, NHB race and low BMI were independent factors associated with increased risks of recurrence with marginal significance. Although the fraction of patients who underwent open surgery was higher among NHB patients, surgical approach was not independently associated with increased risks of recurrence or death based on multivariable analyses. No differences in receipt of GCC were observed among the racial and ethnic groups.

Our findings are consistent with observations made by previous single-center studies that analyzed racial and ethnic disparities in colorectal cancer. The fact that NHB patients tend to have shorter OS has been widely reported (29, 30), but the literature on racial and ethnic disparities and disease recurrence following surgical resection of colon tumors is much sparser. Snyder and colleagues (22) compared recurrence rates and OS between NHB and NHW patients using the National Cancer Database, and a higher risk of recurrence and mortality was observed in NHB patients when compared with NHW patients. In that study, NHB patients had higher percentages of GCC, and NHB race was an independent factor associated with an increased risk of recurrence and shorter survival. Other studies reported no significant differences in outcomes when patients treated at equal-access healthcare systems were stratified by race (31–33). However, some of those studies involved cohorts selected for specific characteristics that may limit the generalizability of their conclusions. For example, Lee and colleagues (31) analyzed data from patients with stage III colon cancer that were all treated with neoadjuvant treatment, whereas the other studies used data from military personnel only, who may not be an accurate representation of the general population in terms of baseline health status and lifestyle (32, 33). In a subgroup analysis using the National Cancer Database, which focused on patients with private insurance, Kamath and colleagues (34) found that NHB patients still had worse OS compared with White patients irrespective of SES, which is consistent with our results.

The ultimate reasons for the association between NHB race and worse outcomes still need to be further elucidated with the use of larger cohorts. The worse outcomes that we observe for the NHB patients could be at least partially explained by racial differences in baseline clinical variables, such as the higher percentages of certain comorbidities in NHB patients despite earlier age of diagnosis and increased proportions of NHB patients with nodal involvement at baseline (stage III disease). This distinct profile of NHB patients has also been reported in previous studies (22, 35). However, the fact that NHB race remains marginally significant in multivariable analysis after controlling for comorbidities, stage, and other social factors emphasizes the need for further investigation in order to identify the roots of this disparity.

Our results should be understood in light of several limitations. First, the number of patients in our study, particularly NHB and Hispanic patients, remains small for comprehensive multivariate analyses. Because of this, some of our group comparisons may suffer from limited statistical power; the lack of statistical significance in some of the differences that we report should be interpreted with caution and revisited as larger sample sizes become available in the future. For example, we observed no significant association between Medicaid usage and outcomes in the multivariate setting, but this might be due to the limited number of Medicaid patients in our cohort (*n* = 53). Second, the population included in this study is not fully representative of the US population; instead, it is strongly determined by the geographic area served by MSKCC, which primarily encompasses the New York City metropolitan region, and only patients who had insurance or could afford to pay for their care at MSKCC were included. Third, patients of racial and ethnic groups beyond NHW, NHB, and Hispanic were excluded from our analysis. These elements, combined with the retrospective design of our study, result in geographic and socioeconomic selection biases that need to be acknowledged. Fourth, a number of unmeasured factors, including potentially relevant social determinants of health (e.g., diet, lifestyle, physical activity, environmental exposures, and cultural beliefs), are missing from our dataset, which may play a role in explaining the differences in clinical outcomes. Lastly, our analyses are observational and correlative in nature. Our results do not imply causation and should be further validated in larger, curated cohorts.

In conclusion, NHB patients had earlier and higher rates of recurrence compared with their NHW and Hispanic counterparts, and this trend was observed even after controlling for demographic and socioeconomic variables. Accordingly, our data suggest that NHB patients might benefit from enhanced postsurgical surveillance for the early detection of recurrences. These results also highlight the need for future studies using larger cohorts to further explore biologic and detailed social or environmental factors driving racial disparities in outcomes of nonmetastatic colon cancer.

## Supplementary Material

Supplementary Data Table S1 Supplementary Table S1. Master Patient Table

Supplementary Data Legends Legends for supplementary data

Figure S1 Figure S1. Kaplan-Meier curves showing recurrence free survival (RFS) from time of surgery for the following variables.

Figure S2 Figure S2. Kaplan-Meier curves show overall survival from time of surgery for NHW, NHB and Hispanic patients.
