# Supplementary material for: Assessing Racial Disparities in Guideline-Concordant Care and Clinical Outcomes after Surgical Resection of Nonmetastatic Colon Cancer at a Comprehensive Cancer Center
Source: Cancer Res Commun. 2025 Jul 18;5(7):1171–9. doi: 10.1158/2767-9764.CRC-24-0633 (PMC12272046; doi:10.1158/2767-9764.CRC-24-0633)
Supplement: Supplementary Data Legends — Legends for supplementary data [file crc-24-0633_supplementary_data_legends_suppsdl.docx]

SUPPLEMENTARY FIGURES

**Figure S1. Kaplan-Meier curves showing recurrence free survival (RFS) from time of surgery for the following variables: A.**Age groups <50, 50-64, and 65+, **B.**Sex, **C.**History of cancer, **D.**ASA physical status classification, **E.**Insurance type, **F.**Number of comorbidities, **G.**BMI category, **H.**Tumor site, **I.**Surgical approach, **J.**Pathologic stage, **K.**Yost class, **L.**Receipt of guideline concordant care.

**Figure S2. A.**Kaplan-Meier curves show overall survival from time of surgery for NHW, NHB and Hispanic patients. **B.**Forest plot summarizes results from multivariate Cox regression analysis for OS. **C.**Forest plot summarizes results from stage-specific multivariate Cox regression analysis for RFS.

SUPPLEMENTARY TABLES

**Supplementary Table 1: Master Patient Table**
