# Supplementary figures and images for: Assessing Racial Disparities in Guideline-Concordant Care and Clinical Outcomes after Surgical Resection of Nonmetastatic Colon Cancer at a Comprehensive Cancer Center
Source: Cancer Res Commun. 2025 Jul 18;5(7):1171–9. doi: 10.1158/2767-9764.CRC-24-0633 (PMC12272046; doi:10.1158/2767-9764.CRC-24-0633)

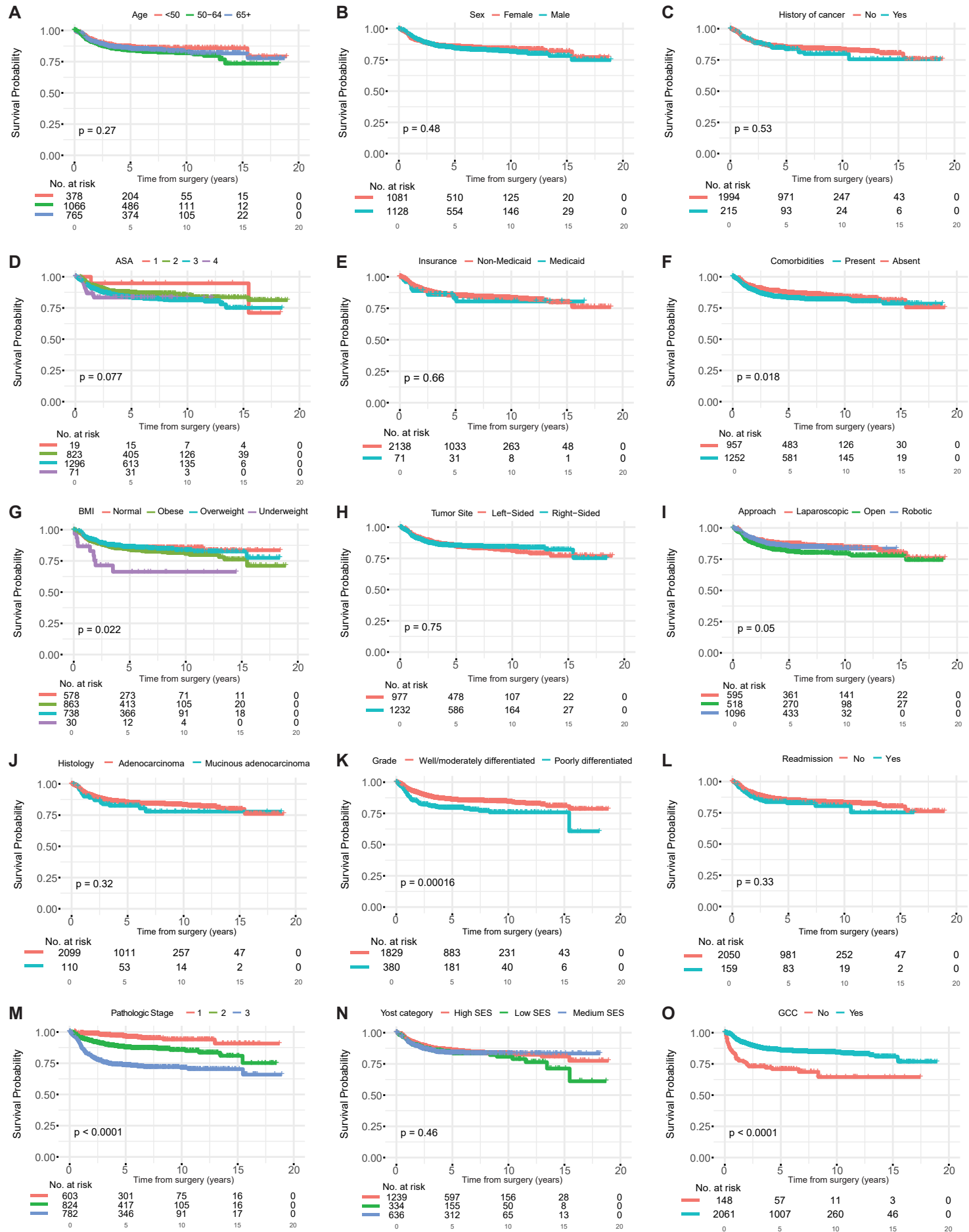

Supplemental Figure 1

Supplement: Figure S1 — Kaplan-Meier curves showing recurrence free survival (RFS) from time of surgery for the following variables. [file crc-24-0633_figure_s1_suppsf1.pdf]
