# Supplementary material for: Assessing Racial Disparities in Guideline-Concordant Care and Clinical Outcomes after Surgical Resection of Nonmetastatic Colon Cancer at a Comprehensive Cancer Center
Source: Cancer Res Commun. 2025 Jul 18;5(7):1171–9. doi: 10.1158/2767-9764.CRC-24-0633 (PMC12272046; doi:10.1158/2767-9764.CRC-24-0633)
Supplement: Figure S2 — Kaplan-Meier curves show overall survival from time of surgery for NHW, NHB and Hispanic patients. [file crc-24-0633_figure_s2_suppsf2.pdf]

A

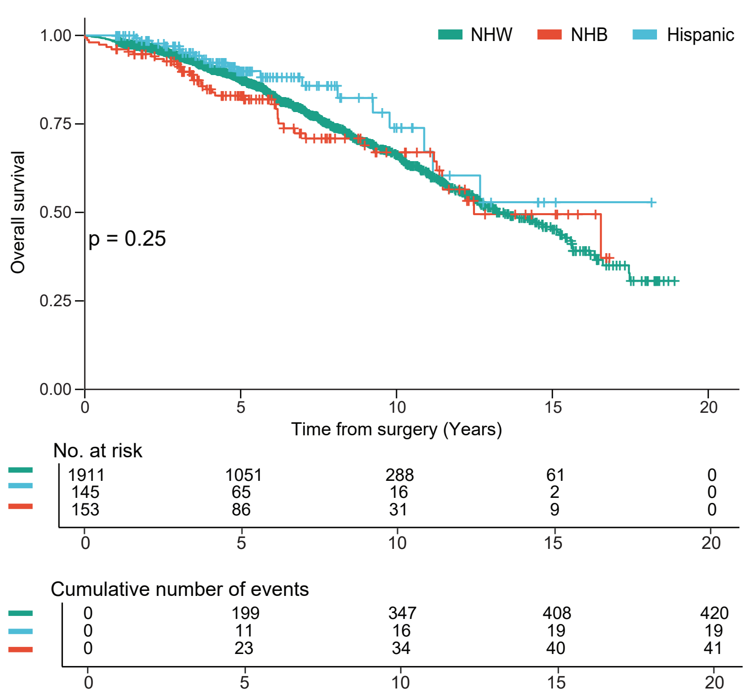

B

Variable		N	Hazard ratio		p
Race	NHW	1911		-	
	Hispanic	145		0.86 (0.54, 1.38)	0.54
	NHB	153		1.26 (0.90, 1.77)	0.18
Age	> 65	1066		-	
	< 50	378		0.23 (0.15, 0.35)	<0.001
	50-64	765		0.26 (0.20, 0.33)	<0.001
Insurance	Non-Medicaid	2138		-	
	Medicaid	71		0.81 (0.46, 1.42)	0.46
Comorbidities	Absent	957		-	
	Present	1252		1.91 (1.50, 2.43)	<0.001
BMI	Normal	578		-	
	Obese	863		0.96 (0.76, 1.22)	0.75
	Overweight	738		0.99 (0.78, 1.26)	0.93
	Underweight	30		1.68 (0.92, 3.07)	0.09
SES category	High SES	1239		-	
	Low SES	334		1.22 (0.96, 1.56)	0.11
	Middle	636		1.09 (0.88, 1.35)	0.43
Approach	Robotic	1096		-	
	Laparoscopic	595		1.30 (1.02, 1.66)	0.03
	Open	518		1.25 (0.97, 1.61)	0.09
Histology	Adenocarcinoma	2099		-	
	Mucinous adenocarcinoma	110		1.01 (0.70, 1.47)	0.95
Grade	Well/moderately differentiated	1829		-	
	Poorly differentiated	380		1.24 (0.98, 1.55)	0.07

0.2 0.5 1 2

C

Variable		N	Hazard ratio		p	N	Hazard ratio		p	N	Hazard ratio		p
Race	NHW	533		-		722		-		656		-	
	Hispanic	29		1.90 (0.40, 8.95)	0.42	58		0.83 (0.33, 2.08)	0.7	58		0.78 (0.44, 1.40)	0.41
	NHB	41		3.52 (1.12, 11.0)	**0.03**	44		1.41 (0.64, 3.12)	0.4	68		1.19 (0.74, 1.93)	0.48
Age	> 65	287		-		459		-		320		-	
	< 50	88		1.95 (0.02, 1.57)	0.12	122		0.78 (0.40, 1.51)	0.5	168		0.96 (0.62, 1.48)	0.85
	50-64	228		0.73 (0.30, 1.78)	0.49	243		0.68 (0.41, 1.13)	0.1	294		1.09 (0.79, 1.52)	0.59
Insurance	Non-Medicaid	587		-		792		-		759		-	
	Medicaid	16		1.73 (0.22, 13.4)	0.60	32		1.03 (0.37, 2.87)	0.9	23		1.08 (0.50, 2.34)	0.84
Comorbidities	Absent	259		-		331		-		367		-	
	Present	344		0.63 (0.18, 2.09)	0.31	493		1.31 (0.81, 2.12)	0.3	415		1.22 (0.88, 1.70)	0.23
BMI	Normal	145		-		236		-		197		-	
	Obese	238		1.34 (0.48, 3.76)	0.58	307		0.93 (0.57, 1.53)	0.8	318		1.21 (0.83, 1.77)	0.32
	Overweight	304		6.13 (0.17, 2.09)	0.43	271		0.79 (0.47, 1.32)	0.4	253		1.10 (0.74, 1.63)	0.63
	Underweight	6		7.47 (1.34, inf)	0.99	10		2.53 (0.74, 8.64)	0.1	14		1.93 (0.81, 4.58)	0.14
SES category	High	361		-		449		-		429		-	
	Low	89		0.98 (0.33, 2.93)	0.86	130		0.87 (0.50, 1.52)	0.6	115		1.27 (0.83, 1.92)	0.27
	Middle	153		0.38 (0.11, 1.34)	0.14	245		0.68 (0.41, 1.10)	0.1	238		1.24 (0.91, 1.70)	0.18
Approach	Robotic	311		-		378		-		407		-	
	Laparoscopic	176		0.77 (0.28, 2.17)	0.61	220		1.15 (0.71, 1.87)	0.6	199		0.77 (0.54, 1.11)	0.16
	Open	116		1.28 (0.45, 3.63)	0.68	226		1.03 (0.63, 1.68)	0.9	176		1.30 (0.94, 1.81)	0.12
Histology	Adenocarcinoma	590		-		780		-		729		-	
	Mucinous adenocarcinoma	13		8.07 (1.24, inf)	0.99	44		1.26 (0.57, 2.77)	0.6	53		0.76 (0.43, 1.36)	0.36
Grade	Well/moderately differentiated	558		-		686		-		585		-	
	Poorly differentiated	45		1.34 (0.30, 5.97)	0.62	138		0.80 (0.46, 1.40)	0.4	197		1.47 (1.08, 2.00)	**0.01**
GCC	Yes	563		-		813		-		685		-	
	No	40		0.47 (0.07, 3.61)	0.57	11		0.74 (0.10, 5.52)	0.8	97		2.30 (1.59, 3.33)	**<0.001**

0.05 0.2 0.5 1 2 5 10 — **Stage I**

0.1 0.2 0.5 1 2 5 — **Stage II**

0.5 1 2 — **Stage III**

Supplemental Figure 2
